# Formulation of the Microbicide INP0341 for *In Vivo* Protection against a Vaginal Challenge by *Chlamydia trachomatis*

**DOI:** 10.1371/journal.pone.0110918

**Published:** 2014-10-30

**Authors:** Christian Pedersen, Anatoly Slepenkin, Sara B. E. Andersson, Jonas H. Fagerberg, Christel A. S. Bergström, Ellena M. Peterson

**Affiliations:** 1 Department of Pharmacy, Uppsala University, Biomedical Center, Uppsala, Sweden; 2 Department of Pathology and Laboratory Medicine, University of California Irvine, Irvine, California, United States of America; University of California Merced, United States of America

## Abstract

The salicylidene acylhydrazide (SA) compounds have exhibited promising microbicidal properties. Previous reports have shown the SA compounds, using cell cultures, to exhibit activity against *Chlamydia trachomatis*, herpes simplex virus and HIV-1. In addition, using an animal model of a vaginal infection the SA compound INP0341, when dissolved in a liquid, was able to significantly protect mice from a vaginal infection with *C. trachomatis*. To expand upon this finding, in this report INP0341 was formulated as a vaginal gel, suitable for use in humans. Gelling agents (polymers) with inherent antimicrobial properties were chosen to maximize the total antimicrobial effect of the gel. *In vitro* formulation work generated a gel with suitable rheology and sustained drug release. A formulation containing 1 mM INP0341, 1.6 wt% Cremophor ELP (solubility enhancer) and 1.5 wt% poly(acrylic acid) (gelling and antimicrobial agent), was chosen for studies of efficacy and toxicity using a mouse model of a vaginal infection. The gel formulation was able to attenuate a vaginal challenge with *C. trachomatis,* serovar D. Formulations with and without INP0341 afforded protection, but the inclusion of INP0341 increased the protection. Mouse vaginal tissue treated with the formulation showed no indication of gel toxicity. The lack of toxicity was confirmed by *in vitro* assays using EpiVaginal tissues, which showed that a 24 h exposure to the gel formulation did not decrease the cell viability or the barrier function of the tissue. Therefore, the gel formulation described here appears to be a promising vaginal microbicide to prevent a *C. trachomatis* infection with the potential to be expanded to other sexually transmitted diseases.

## Introduction

A group of salicylidene acylhydrazide (SA) compounds have exhibited promising microbicidal properties in a number of recent studies [1], [2], [3]. The mechanism of action of the SA compounds is not completely understood but directly or indirectly involves iron, in that iron can reverse the antimicrobial activity [4]. Of the SA compounds INP0341 has been the most extensively studied, and along with other SA compounds has been shown *in vitro* to exhibit activity against a variety of sexually transmitted organisms, including *Chlamydia trachomatis,* herpes simplex virus and HIV-1 [1], [2], [3], [4], [5]. In the case of HIV, the compounds exhibited IC_50_ values (50% inhibition of virus) as low as 1 µM, while at the same time showing promise from a toxicological point of view [1]. The compounds have also been shown to be effective *in vivo*, when tested as a non-formulated solution in a mouse model to protect against a vaginal challenge with *C. trachomatis* [6]. Therefore, these compounds are promising candidates for use as a vaginal microbicide. The next logical step in the development of a vaginal microbicide using these compounds is to formulate them for eventual use in humans. In this present study we focused on the SA compound INP0341.

Typical formulations intended for vaginal use are gels, ointments and creams. A crucial property of a vaginal microbicide is complete coverage of the vaginal mucosa and this is dependent on several aspects, including rheological properties of the formulation and the administration interval. The rheological properties determine the gel spreading rate over the mucosa, as well as the time retained in the vaginal tract [7]. A formulation of low viscosity will have a short residence time, most likely demanding frequent administration of the formulation. For this reason many of the gels developed for vaginal administration are semi-solids, pseudoplastic materials of high resting viscosity. Vaginal gels typically consist of the polymer dissolved in a slightly acidic buffer (pH ∼4.0–6.0) to comply with the pH of the vaginal tract [8], [9]. Common polymers are poly(acrylic acid) (PAA) and hydroxyethyl cellulose (HEC), sometimes used in combination [9]. Previous studies have suggested that Carbopol 974P NF alone, which is a PAA type polymer, mediates some protection against *C. trachomatis* and several other sexually transmitted diseases (STDs) [10]. In addition to its possible microbicidal effect, PAA polymers are strongly mucoadhesive, stronger than the universal placebo gel HEC, and therefore the resulting increased residence time on the mucosa further motivates the choice of PAA as gelling agent for such gels.

The long-term objective of this study was to formulate the poorly soluble SA compound INP0341 as a vaginal gel formulation that could be administered for continuous release of INP0341 for a prolonged period so in the future it could protect women against viral and bacterial STDs. A challenge with this work was to find a suitable solvent for INP0341 for which Cremphor ELP was ultimately employed. The parameters used in the development of the formulation described here were: the need to solubilize a sufficient concentration of INP0341; have suitable rheology to ensure high coverage of the vaginal mucosa and long contact times; and the release of INP0341 *in vivo* over at least 24 h with the goal of increasing patient compliance. We present the properties of the INP0341 formulation developed using these parameters, and the microbicidal effect obtained when tested in a well-established mouse model of a vaginal challenge with a common human *C. trachomatis* serovar [11], [12].

## Materials and Methods

### Organisms and cell lines

*C. trachomatis*, serovar D (UW-3), *Lactobacillus jensenii* strain 6G (25258), *Lactobacillus crispatus* (33197) and HeLa 229 cells were obtained from the American Type Culture Collection (ATCC) (Manassas, VA, USA). HeLa cells were grown in Eagle’s minimal essential medium (MEM) (Gibco, Invitrogen Corporation, Grand Island, NY, USA) supplemented with 5% fetal bovine serum (FBS, Atlanta Biologicals, Lawrenceville, GA, USA), 2 mM L-glutamine (Meditech, Herndon, VA, USA), and 50 µg/ml of gentamicin (Meditech) (MEM-FBS). *C. trachomatis* stocks were raised in HeLa cells as previously described [13]. *Lactobacillus* species were maintained on 5% sheep blood agar (Becton Dickinson, Frankin Lakes, NJ, USA) and Lactobacillus MRS agar (Oxoid, Basingstoke, England). All cultures were incubated at 37°C in 5% CO_2_ for 48 h. Organisms were sub-cultured twice prior to being used in an assay.

### Preparation and characterization of the vaginal gels

#### INP0341

The compound INP0341 was synthesized from 5-chloro-2-hydroxy-3-methylbenzaldehyde and 2,4-dihydroxybenzhydrazide and the product was confirmed by nuclear magnetic resonance (NMR) and mass spectroscopy (MS) as previously described [14].

The melting temperature (T_m_) of INP0341 was examined using Differential Scanning Calorimetry (DSC6200, Seiko Japan). 1.0 mg of powder was added to an aluminum pan and covered with a pierced lid. The sample was flushed with nitrogen and heated at a rate of 10°C per minute up to 300°C. The resulting thermogram was used to determine T_m_.

Lipophilicity expressed as the partition coefficient between octanol and water (logP) and dissociation constants (pKa) were determined with the T3 platform from Sirius Analytical Instruments (Forest Row, UK). The electrode was calibrated using a blank titration from pH 1.8 to 12.2. For the pKa measurement, a 10 mM standard dimethyl sulfoxide (Sigma Aldrich, Sweden) solution was made. Thereafter, 5 µL of the standard solution was added to 25 µL phosphate buffer. The T3 instrument added a predetermined volume of 0.15 M KCl, followed by a pH-metric titration from low to high pH using 0.5 M NaOH. During the titration, the T3 instrument was collecting a UV/Vis spectrum by using D-PAS technique to establish a titration curve. Octanol (Sigma Aldrich, Sweden) was used for logP measurements. The measurements were performed at 37°C±1°C, and under argon to minimize the effect of dissolved CO_2_.

The solubility of INP0341 in MilliQ water and simulated vaginal fluid (SVF) was determined using a shake flask method [15], [16]. An excess amount of compound was added to 1 mL of each solvent, and the compound was allowed to dissolve during shaking for 48 h (after which equilibrium was reached). The samples were then centrifuged for 15 min at 10,000×*g* at the same temperature as during the solubility experiment. Samples were taken from the supernatant in triplicate, and diluted with ACN:H_2_O (50∶50 by volume). Dissolved concentrations were determined by LC-MS/MS.

The diffusion coefficient of INP0341 was calculated using the ADMET predictor 5.5 (Simulations Plus, CA, USA).

#### Gel formulation of INP0341

The polymers Polycarbophil (Noveon AA-1 Polycarbophil) and Carbopol (Carbopol 974P NF) were obtained from Lubrizol, Belgium (both polymers are of USP/NF grade). Bovine serum albumin (>98%), Cremophor ELP, glucose anhydrous (>98%), acetic acid (>99%), lactic acid, poly(ethylene glycol) (PEG; Mw 400), urea, sodium chloride (>99%), potassium hydroxide (puriss. p.a.), calcium hydroxide (puriss. p.a.) and glycerol (>99%) were obtained from Sigma-Aldrich, Sweden. Sodium hydroxide solution (5 M) was obtained from VWR, Sweden. Ultrapure MilliQ water was used throughout the study.

For gels containing 0.2–1.0 mM INP0341: 10 g of INP0341 pre-solution was prepared by initially adding 3.14–15.7 mg INP0341 to 1.0 g of Cremophor ELP, which served to enhance the solubility of INP0341, and 0.25 g of PEG 400, and dissolving it during 4 h at 60°C. Water was thereafter slowly added to the mixture during magnetic stirring. After water addition, the solution was stirred at room temperature for additional 30 min. The INP0341 pre-solution was stored in the refrigerator until use.

The gels were produced using a mortar, pistil and scrape cards. For a final gel weight of 30 g: 1.5 g of glycerol and 20 g of water was mixed in a mortar. 0.1–0.2 g of Carbopol and 0.2–0.4 g of Polycarbophil was then added, to achieve gels with a total polymer concentration of 1.0–2.0 wt% using a Carbopol:Polycarbophil weight ratio of 1∶2. The mixture was stored at 8°C overnight for swelling and dissolution of the polymers. 1.2–6.0 g of INP0341 pre-solution was then added (for a final Cremophor ELP concentration of 0.4–2.0 wt%; the Cremophor ELP:PEG ratio was 4∶1 in every gel) followed by mixing for 10 minutes. For the production of a control gel without INP0341 and Cremphor ELP, the INP containing pre-solution was replaced by MilliQ water. Sodium hydroxide, 5 M, was then added to reach a final gel pH of 5.2±0.1 (e.g. 410 µl of 5 M NaOH for a 30 g gel with 1.5 wt% polymer) followed by 5 minutes of mixing. Water was added to roughly 1 gram more than the final weight, and the gel was mixed for approximately 30 minutes during water evaporation, until the final weight was reached. The gels were stored at 4°C until use. The gel pH was measured with a Cyberscan pH 510 pH-meter (Eutech Instruments, Sweden), equipped with an Orion 8103 pH-electrode (Thermo Scientific, Sweden). A Vapro 5520 Osmometer (Wescor, USA) was used to measure the osmolality of the INP0341 gel (containing 1.5 wt% PAA and 1.6 wt% Cremophor ELP) and for comparison the commercially available vaginal gels Crinone (8% progesterone) and Replens (vaginal moisturizer) were used. Osmolality results represent the average and standard deviation of triplicate measurements.

#### Rheological characterization of the gels

Rheological measurements were performed using a Bohlin VOR controlled rate rheometer (Bohlin Reologi, Lund, Sweden) of the coquette type. Gel samples with 1–2 wt% PAA were characterized, as well as the commercially available vaginal gels Crinone and Replens. A concentric cylinder (C14) measuring system was used. After being loaded in the measuring cylinder, the samples were centrifuged for 3 minutes at 1,500×*g* to remove entrapped air.

Strain sweeps (0.00135≤γ≤0.182) were performed at a frequency of 1 Hz to determine the linear viscoelastic region for each sample. Every sample exhibited a critical strain (γ_crit_), i.e. a strain at which the elastic modulus (G’) started to decrease. The elastic modulus in the linear region was defined as G’_lin_, and the strain value at 0.9×G’_lin_ was used as γ_crit_. The yield stress of each sample was calculated, see Eq. 1, where *δ* is the phase angle.
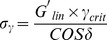
(1)


Shear stress and viscosity were determined by rotational viscometric measurements, using a shear rate interval of 1.2–73.2 s^−1^ [17]. Data were collected over a sweep of lowest to highest shear rate, and results are reported as the mean and standard deviation of triplicate measurements. The gels exhibited yield stresses, and it was therefore suitable to fit shear stress data to a Herschel-Bulkley constitutive equation, see Eq. 2, where *σ* is the shear stress, *m* is the consistency index and *n* is the shear-thinning index [7].
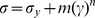
(2)


Yield stress (σ_y_) was determined by strain sweep measurements as described above, while the parameters *m* and *n* were determined by fitting data to the model and using the method of least squares.

To obtain an estimate for the gel spreading rate over vaginal mucosa, and to support the choice of polymer concentrations for the gels to be used *in vivo*, a theoretical squeezing flow model was employed, as described in the Supporting Information (Figure S1 in File S1).

#### Stability testing

The chemical stability of INP0341 and rheological stability of the gels were investigated. A gel containing 1 mM INP0341, 1.5 wt% PAA and 2.0 wt% Cremophor ELP was divided into three samples, and stored at 8, 20 and 40°C. The rheology of the three samples was measured after 0, 1 and 8 months, using a Bohlin rheometer as described above. The INP0341 concentrations of the three samples were measured after 8 months of storage using LC-MS/MS.

#### *In vitro* drug release

*In vitro* drug release was investigated using 9 mm Franz cells with a 5 ml receptor volume (PermeGear, Hellertown, PA, USA), together with 5 µm Whatman Cyclopore membranes (Sigma-Aldrich, Sweden). Two samples (each containing 1 mM INP0341) were investigated: a gel (containing 1.5 wt% polymer and 2.0 wt% Cremophor ELP) and a DMSO solution (50 wt% DMSO in 25 mM acetate buffer of pH 5.2). SVF without albumin, which was excluded to avoid problems during LC-MS/MS analysis, was produced as described previously {15}, and used as receptor fluid in the case of drug release from the gel. To study diffusion of INP0341 unaffected by Cremophor ELP micelles, drug release from a 50% DMSO solution was studied using 50 wt% DMSO in 25 mM acetate buffer (pH 5.2) as receptor fluid. Experiments were started by adding 0.50 g of sample to each donor chamber. Samples were taken from the receptor chamber during 24 h, and analyzed using LC-MS/MS. The release experiments were performed in triplicate, at 37°C, and mean values and standard deviations are reported.

#### Analytical Methods

A ThermoFinnigan TSQ Quantum Discovery triple-quadropole (electrospray ionization) coupled to a Waters Acquity UPLC was used for concentration determination of the samples obtained during solubility and release experiments. Separation was done on a Waters BEH C18 column, 2.1×50 mm (1.7 µm). Mobile phase A consisted of 5% MeCN in water (0.1% formic acid) and mobile phase B 100% MeCN (0.1% formic acid). The chromatographic run was 2.5 minutes in total and started with 5% of solvent B, followed by a linear gradient from 1.0 to 1.5 min ending at 90% of solvent B, followed by a hold from 1.5 to 1.7 minutes and a linear gradient to return to the initial conditions at 1.8 minutes. The flow rate was 0.5 ml/min and the injected volume was 3 µL.

### Toxicity assay using EpiVaginal tissue (VEC-100)

EpiVaginal tissues (VEC-100) were purchased and used according to the protocols of the manufacturer (MatTek Corporation, Ashland, MA, USA). All tissues were initially transferred to assay medium and incubated at 37°C and 5% CO_2_ overnight, to recover after shipping. Tissues were then exposed to three types of gels, all containing 1.5 wt% PAA polymer: (1) control gel without Cremophor ELP and INP0341 with MilliQ water used as pre-solution in the production of this gel, (2) same as the control gel but also containing 1.6 wt% Cremophor ELP and (3) same as control gel but also containing 1.6 wt% Cremophor ELP and 1 mM INP0341. MilliQ water was used as negative control and 1.0% Triton X-100 was used as positive control as suggested in the manufacturer VEC-100 protocol. Each tissue was exposed to 100 µL of one test article (gel or control), and thereafter incubated for either 4 h or 24 h at 37°C and 5% CO_2_. The positive controls were incubated 45 minutes and 2 h as suggested by the manufacturer. One set of tissues was used for the MTT assay, and another set of tissues was used for TEER measurements. Triplicate tissues were used for each test article and incubation time, and all results are given as mean and standard deviation.

Tissue viability was monitored using the MTT assay as described in the VEC-100 protocol. MTT was extracted from each tissue overnight (in the dark, at room temperature), prior to optical density (OD) measurement at 570 nm. The viability after 4 h and 24 h of exposure of the tissue to the gels was determined by normalizing to negative controls (4 h and 24 h, respectively), using the equation:
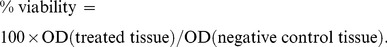
(3)


Transepithelial electrical resistance (TEER) was used to assess changes in tissue barrier function, using a Millicell ERS system (Millipore, Billerica, MA, USA). TEER was measured before and after sample exposure for each individual tissue, and calculated as:
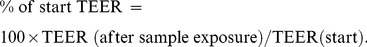
(4)


### Minimum inhibitory concentration (MIC) of *Lactobacillus*

In order to determine the effect of the formulations on normal vaginal flora, *L. jensenii* and *L. crispatus* were used as markers of normal vaginal flora since they represent two of the most common hydrogen peroxide producing *Lactobacillus* species present in normal human vaginal samples. Two-fold serial dilutions of the gels without INP0341 as well as gels formulated to contain 1 mM of INP0341 were made in MRS broth (Oxoid, Hampshire, UK) in a 96-well microtiter plate. The dilutions which ranged from 1∶2 up to 1∶128, corresponded to 500 µM to 7.8 µM of INP0341 in the formulated gel. A sterility check of the gels and broth, and a growth control of the bacterial inoculum, were included in each assay. A standardized suspension of bacteria of 1×10^5^ colony forming units (CFU)/ml in MRS broth was made from an overnight culture grown on MRS agar. Each microtiter well, except those representing the sterility controls, was inoculated with 0.1 ml of the standardized suspension, i.e. 1×10^4^ CFU. Plates were incubated at 37°C in 5% CO_2_ for 24 h and were subsequently examined for turbidity or a visible pellet. In addition, the well with the lowest concentration of gel showing no growth was subcultured onto MRS agar or when all wells showed growth then the well containing the highest percentage of gel was subcultured onto MRS agar plates and incubated as above until visible colonies could be enumerated. The MIC was defined as the lowest concentration of the compound that prevented macroscopic growth.

### Animal Model

A mouse model, previously described, was used to test the ability of the gel formulation to attenuate a genital infection by *C. trachomatis,* serovar D [6]. Six-to-seven-week-old female C3H/HeJ (H-2^k^) mice (Jackson Laboratories, Sacramento, CA, USA) received two subcutaneous doses of 2.5 mg/mouse of medroxyprogesterone acetate (SICOR Pharmaceuticals Inc., Irvine, CA, USA) on days 10 and 3 before a vaginal challenge with *C. trachomatis*. Three groups of mice received a challenge with *C. trachomatis*: the sham treated positive infection control group; one group treated with the gel without INP0341; and one treated with the gel formulated with INP0341. Mice were treated intravaginally with 0.05 ml of gel 24 h and 12 h before challenge, 1 h after challenge and at 12 h intervals up to 5 days after the vaginal challenge. Mice were inoculated on day 0 with 5×10^2^ inclusion forming units (IFU) of *C. trachomatis*. Vaginal swabs were collected twice a week for a month after infection. Specimens were cultured, stained and evaluated as previously described [13]. The lower limit of detection of the vaginal culture was 4 IFU/culture. Cultures with no IFU were considered negative for the purposes of data analysis. The experiments were repeated on two separate occasions with 5 mice in each of the positive control groups and 10 to 15 mice in the experimental groups.

### Histopathology

Mice were given two subcutaneous injections of 2.5 mg of medroxyprogesterone acetate (SICOR) ten and three days as described above. One pair of control mice were sacrificed immediately before all other mice received their first treatment with the vaginal gel and served as the day 0 controls. Pairs of mice were treated with 0.05 ml of gel without or with INP0341, at 12 h intervals for 5 days, and were sacrificed at 24 h intervals. Upon sacrifice the vaginal tissue was harvested intact, cut into thirds as to retain the tubular structure, and immediately immersed in formalin and held until processed. Tissue was embedded in paraffin, sectioned and stained with hematoxylin-eosin.

### Ethics Statement

This study protocol was approved by the Institutional Animal Care and Use Committee of the University of California Irvine (IACUC protocol number 2009-2868). All work was carried out in strict accordance with the recommendations in the Guide for the Care and Use of Laboratory Animals of the National Institutes of Health and all efforts were made to minimize suffering and discomfort.

### Statistical analysis

Fisher’s exact test and Mann-Whitney statistical analysis were used to determine differences between groups of mice regarding the number of mice infected and number of IFU shed per mouse in each experimental group. Data were analyzed by using Sigma Stat 3.5 software (SYSTAT Software Inc., Richmond, CA, USA). A P-value of <0.05 was considered significant.

## Results

### Characterization and formulation of INP0341

The thermogram from the melting temperature (T_m_) measurement showed no indications of solvent residues or polymorphic material, and the T_m_ of INP0341 was determined to be 273°C. The octanol/water partition coefficient (logP) was determined to be 4.37. Hence the compound was proven to belong to the high melting and highly lipophilic compound group. For such compounds water solubility is typically very low as a result of the high crystal lattice energy limiting dissociation from the solid state and the high lipophilicity limiting hydration. In addition, measurements of dissociation constants showed that INP0341 has acidic pKa values of 6.72, 9.07 and 10.37. INP0341 will therefore be uncharged in the gel designed in the present work (pH 5.2), and in the acidic environment of the vagina (pH ∼4–6). Therefore, the solubility in the vaginal tract will be equal to the intrinsic solubility (lowest solubility in the pH-dependent solubility curve) and no positive solubility effects in response to the pH-dependency can be expected. The solubility of INP0341 was determined to 2.2±0.8 µM in MilliQ water (20°C) and 3.1±0.6 µM in SVF (37°C). This low solubility is a significant challenge when developing a therapeutic and clinically relevant formulation. To address this challenge we used the commonly used surfactant Cremophor ELP which is an approved excipient in e.g. the vaginal gel KY Plus (Johnson and Johnson, New Brunswick, NJ). With this approach the solubility increased to at least 1 mM when 1.6 wt% Cremophor ELP was used in the gels. The diffusion coefficient of INP0341 in water was calculated to be 8.6×10^−6 ^cm^2^/s and was used during release studies to determine to which extent INP0341 was diffusing as monomers or after being solubilized in Cremophor ELP micelles.

### Rheology

**Figure 1:**
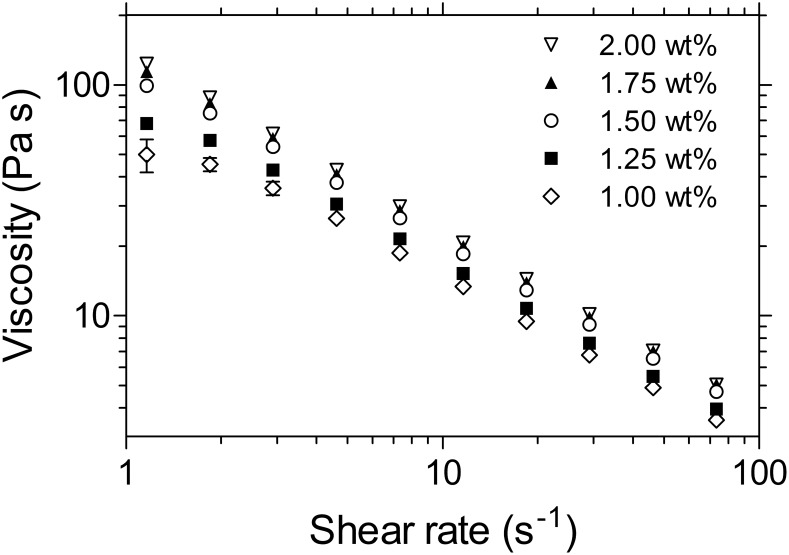
Gel viscosity as a function of shear rate. Gels with PAA concentrations of 1.0–2.0 wt% were measured by continuous rotational viscometry at 37°C.

Figure 1 shows the viscosity as a function of shear rate for gels with 1.0–2.0 wt% PAA. The reported viscosities are the steady shear viscosities, calculated from the torque measured after 1 minute of continuous shear. All gels exhibit shear thinning rheology, i.e. decreasing viscosity with increasing shear rate, which is a warranted feature of vaginal gels [18]. The viscosity values of the PAA gels are within the viscosity range of commercial vaginal gels [18]. The elastic modulus as function of strain (Figure S1 in File S1) and the shear stress as function of shear rate (Figure S2 in File S1) are reported in the Supporting Information. All PAA gels exhibited yield stress, i.e. positive shear stress values at zero shear rate, as shown in Table S1 in the File S1. The yield stress of the INP0341 gel studied *in vivo* (1.5 wt% PAA) was determined to be 11.0 Pa, which was slightly lower than observed for the commercial vaginal gels Crinone (16.2 Pa) and Replens (14.3 Pa). The rheological data was used in combination with a squeezing flow model, after which 1.5 wt% PAA was chosen as a suitable polymer concentration for *in vivo* use (File S1).

### Osmolality and pH of the gel formulation

The INP0341 gel chosen for efficacy and toxicity studies (containing 1.5 wt% PAA, 1.6 wt% Cremophor ELP and 1 mM INP0341) had an osmolality of 658±6.0 mmol/kg. In comparison, the osmolalities of the commercial gels Replens and Crinone were 1718±9.0 and 1901±14.3 mmol/kg, respectively. The INP0341 gels had a pH of 5.2±0.1.

### Stability of the formulation

**Figure 2:**
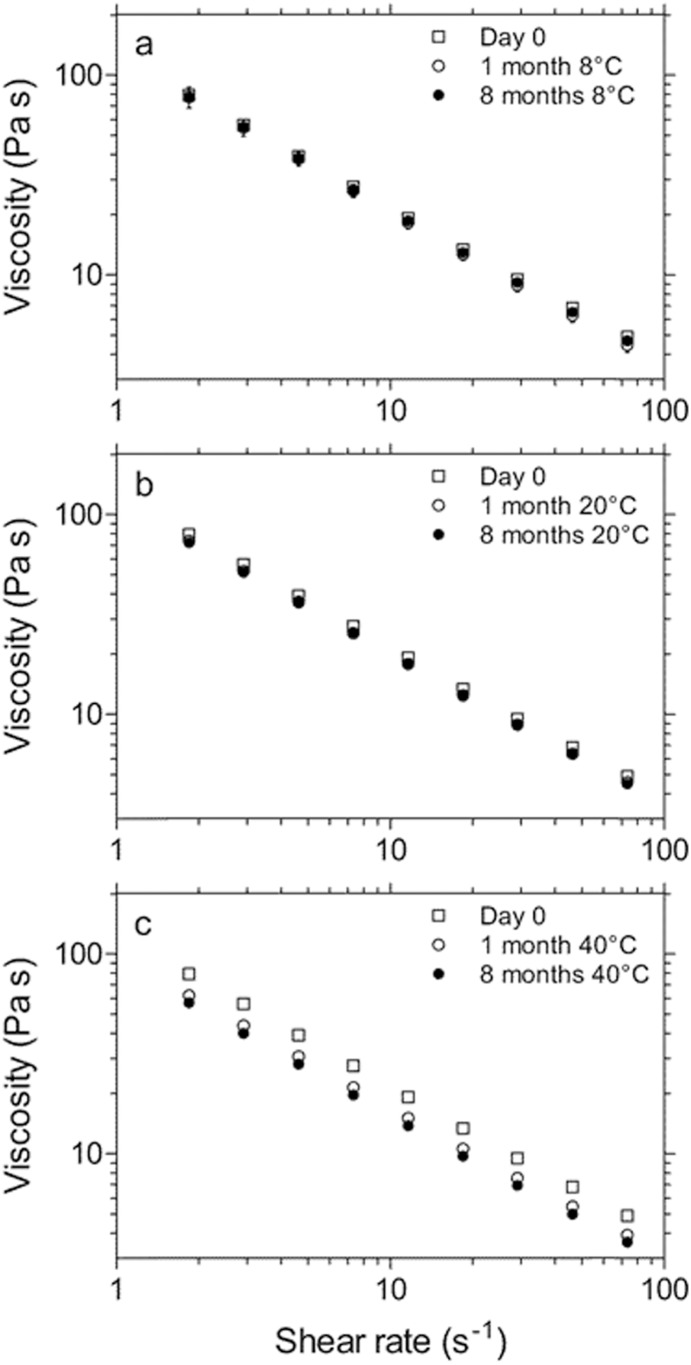
a–c. Rheological stability of the gel formulation containing 1.5 wt% PAA. Measurements were performed by continuous rotational viscometry at 37°C, after 0, 1 and 8 months of storage. The gels were stored at (a) 8°C, (b) 20°C and (c) 40°C.

A gel containing 1 mM INP0341, 1.5 wt% PAA and 2.0 wt% Cremophor ELP was divided into three samples, and stored at 8, 20 and 40°C. After 8 months of storage, the INP0341 concentrations of the three samples were 978±59 µM (8°C), 947±42 µM (20°C) and 853±25 µM (40°C). The rheology of the formulation was stable during storage at 8°C and 20°C, while a decrease in viscosity was observed after storage at 40°C (Figure 2).

The Cremophor ELP concentration was slightly higher in the gel for stability tests (2.0 wt% Cremophor ELP) as compared to the gels for efficacy and toxicity tests (1.6 wt% Cremophor). During the progress of the project, it was observed that the Cremophor ELP concentration could be lowered from 2.0 wt% to 1.6 wt%, while maintaining complete solubility of 1 mM INP0341. The Cremophor ELP concentration was therefore lowered to 1.6 wt% for the efficacy and toxicity studies, to minimize potential toxicity of the formulation. The difference in Cremophor ELP concentration (1.6 wt% vs. 2.0 wt%) is not expected to have any effect on the stability of INP0341 in the gel, since the drug will be completely dissolved in Cremophor micelles in both cases. The rheological (PAA) stability is also not expected to be affected by differences in Cremophor concentration; PAA degradation is an oxidative process, and nonionic surfactants such as Cremophor ELP do not have any influence on such degradation [19].

### *In vitro* drug release

**Figure 3:**
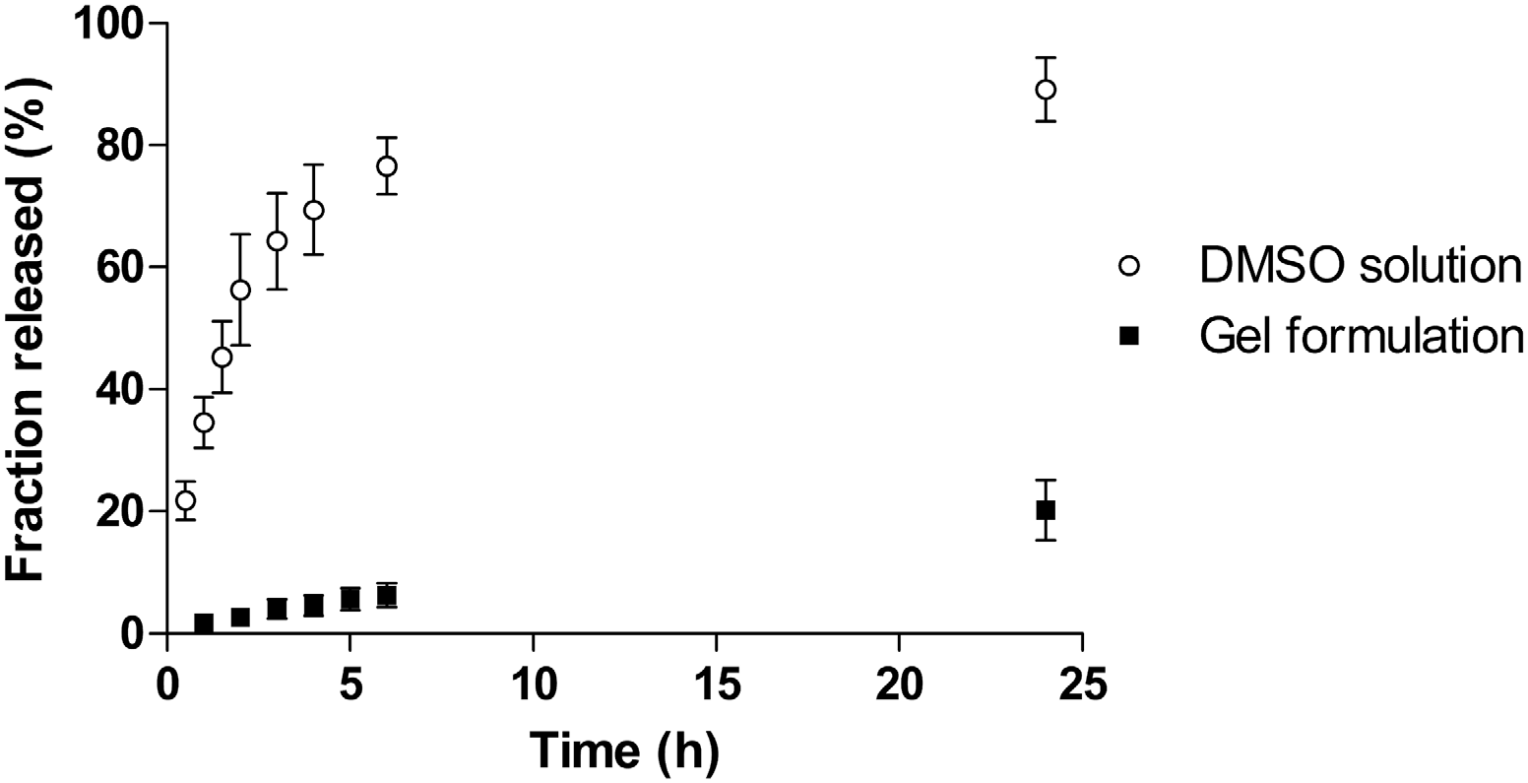
*In vitro* drug release. Comparison of *in vitro* INP0341 release from the gel formulation and from a 50% DMSO solution. A sustained release is observed from the gel, when INP0341 is solubilized in Cremophor ELP micelles.

The release of INP0341 from the gel formulation was studied *in vitro* using Franz cells, to confirm that the gel formulation yields slow drug release. SVF without albumin was used as receptor liquid. The drug release was expected to be governed by Cremophor ELP diffusion, since INP0341 is solubilized in Cremophor ELP micelles in the gel. For comparison, INP0341 release was also studied in the absence of micelles, using 50 wt% DMSO in 25 mM acetate buffer (pH 5.2) as both dissolution media and receptor liquid. Figure 3 shows that the drug release is considerably slower from the gel, as compared to the DMSO solution. After 2 h, 56% of the dose has been released from the DMSO solution while only 2.7% has been released from the gel formulation. The Cremophor ELP-containing gel formulation therefore contributes to a sustained drug release (roughly 20 times slower) which corresponds well with the expected; the theoretical diffusion coefficient of INP0341 is 8.6×10^−6 ^cm^2^/s (calculated using ADMET predictor 5.5), while the diffusion coefficient of a drug-containing Cremophor ELP micelle is around 0.4×10^−6 ^cm^2^/s [20].

### Toxicity assay using EpiVaginal tissue (VEC-100)

**Figure 4:**
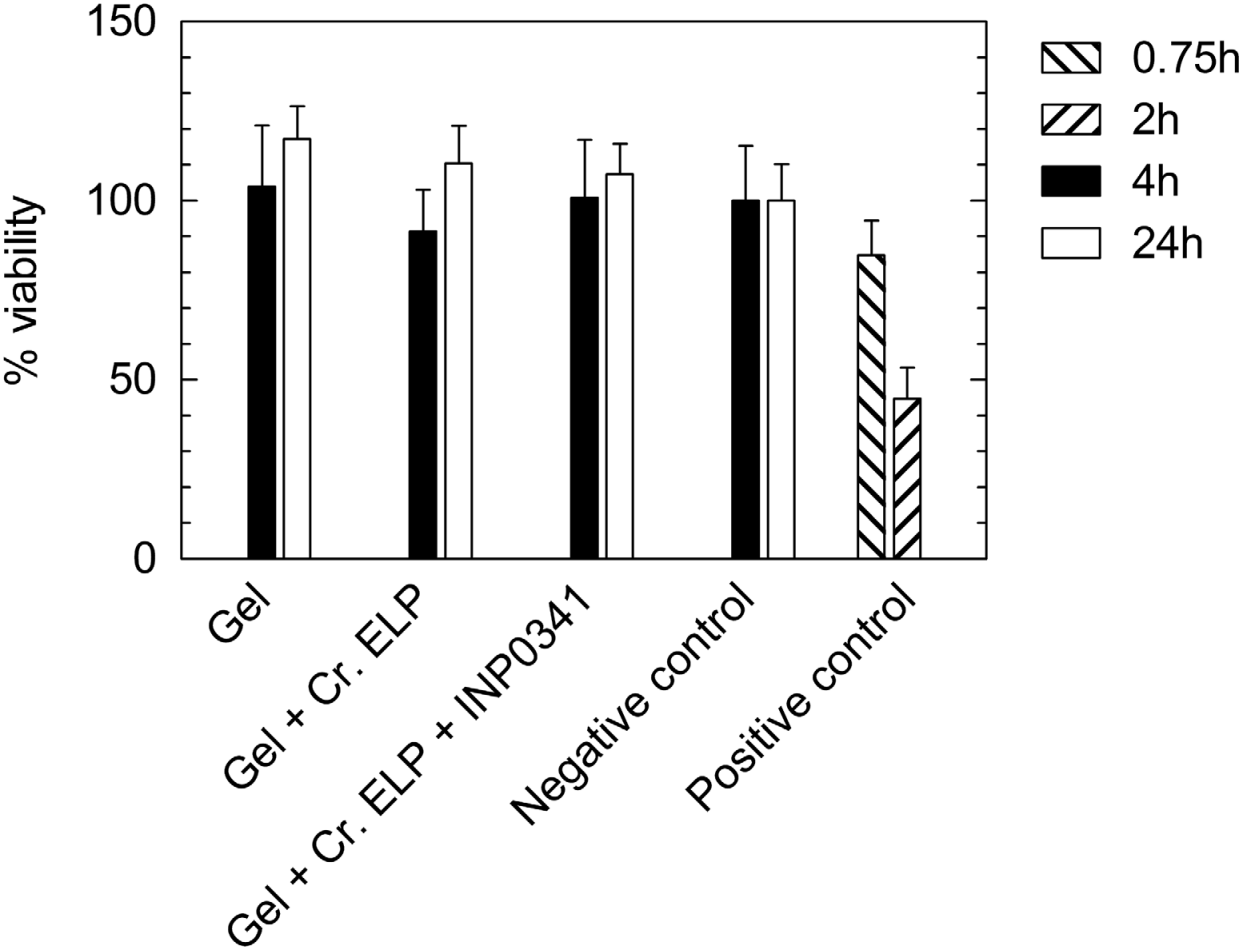
The viability of EpiVaginal tissues after gel exposure, obtained by an MTT assay. The tested gels were (i) gel without Cremophor ELP and INP0341, (ii) gel with 1.6 wt% Cremophor ELP and (iii) gel with 1.6 wt% Cremophor ELP and 1 mM INP0341. MilliQ water and 1.0% Triton X-100 were used as negative and positive controls, as outlined in the EpiVaginal VEC-100 manufacturer’s protocol.

The viability of EpiVaginal tissues after gel exposure, as determined by a MTT assay, is shown in Figure 4. No significant difference in tissue viability could be observed when comparing gels with and without 1.6 wt% Cremophor ELP and 1 mM INP0341. Tissue viability was not reduced by exposure to any of the gels for 4 h or 24 h, as compared to negative control tissues.

TEER measurements were carried out to assess the tissue barrier function, before and after exposure to either gel or control. The TEER results suggest that the gels do not decrease the tissue barrier function, and are therefore consistent with the viability results.

### MIC of the vaginal gel against *Lactobacillus* species

The formulated gel with and without 1 mM INP0341 was tested against *L. jensenii* and *L. crispatus*, two of the most common hydrogen peroxide producing lactobacilli found in the normal human vaginal flora. Two-fold dilutions ranging from 1∶2 to 1∶128 of the formulated gels with and without 1 mM INP0341 were made in MRS broth. For the formulated gel this dilution range corresponded to 500 µM to 7.8 µM of INP0341. The MIC of the formulated gel for both *Lactobacillus* spp. was >500 uM INP0341. The gel not containing INP0341 similarly did not inhibit the growth of the two *Lactobacillus* spp. at any of the dilutions tested. Therefore, the gels had no effect on the growth of these bacterial markers of human normal vaginal flora.

### Effect of the gel formulations in a mouse genital infection model

**Figure 5:**
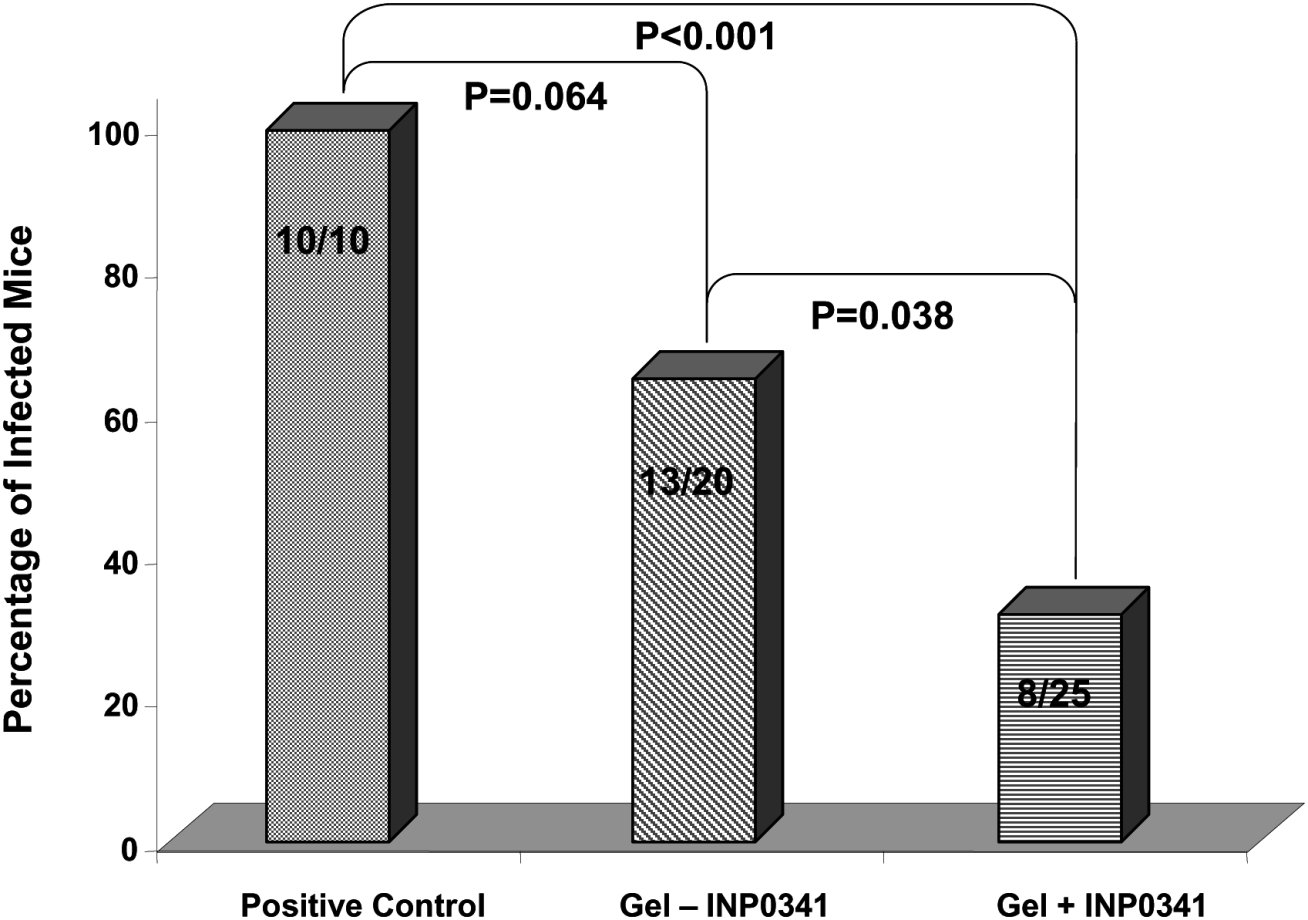
Comparison of the three treatment groups as to protection against a vaginal challenge with *C. trachomatis*. The overall mice culture positive over a month period in each treatment group after a vaginal inoculation with 500 IFU of *C. trachomatis* is shown. The positive control group was sham treated whereas the other two groups received the vaginal gel formulated with and without INP0341. The P values, as determined by the Fischer’s exact test, are shown comparing groups to one another. A P value <0.05 was considered statistically significant. The experiment was repeated with two sets of mice and the graph reflects the combined results of the two experiments.

The formulated gel with and without 1 mM INP0341 showed a protective effect in mice when administered intravaginally, with the former showing a greater effect, against a vaginal challenge with *C. trachomatis*, serovar D (Figure 5). All (10/10) of the mice in the positive control group were infected as shown by positive vaginal cultures within the four weeks after inoculation. In contrast, 65% (13/20) of the mice treated with the gel without INP0341 were infected (P = 0.064) whereas only 32% (8/25) of mice treated with the gel formulation with INP0341 were infected as determined by a positive vaginal culture (P<0.001). The difference between the group of mice treated with the gel not containing INP0341 when compared to mice treated with the formulated INP0341 was significant, in terms of the total number of infected mice over the 4 week observation period (P = 0.038).

**Figure 6:**
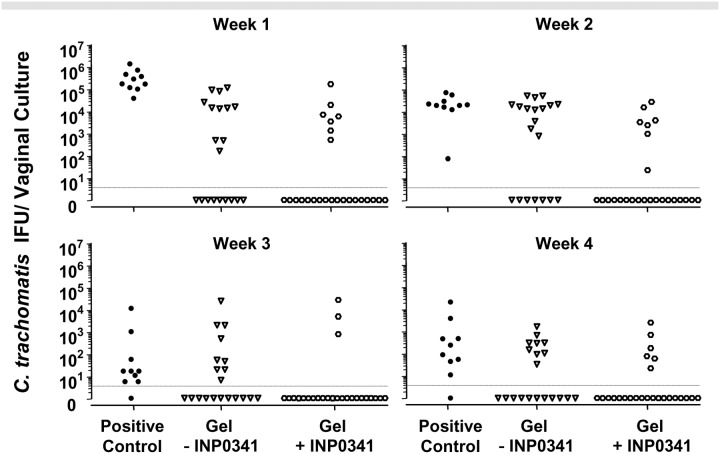
Vaginal culture results for all mice in the three treatment groups. Three groups of mice, positive sham treated controls, mice treated with the gel formulation minus INP0341 and mice treated with the INP0341 formulated gel, were infected with 500 IFU of *C. trachomatis* and followed for 4 weeks by vaginal culture. The number of IFU per vaginal culture/mouse is represented for each week of observation. With the exception of the first week where the infected mice in the positive control group shed higher numbers of *Chlamydia*, regardless of group, infected mice shed similar amounts of *C. trachomatis*.

The infectious burden of *C. trachomatis* as expressed as the yield of IFU/vaginal culture per week for the mice infected is displayed in Figure 6. For the week following the vaginal inoculation of *C. trachomatis*, the number of IFU shed in the culture positive mice was significantly higher in the control group when compared mice treated with the gel without INP0341 and with the gel formulation containing INP0341 (P<0.001). However, regardless of treatment group, it appears that once a mouse was infected as determined by a positive vaginal culture in the first week, with few exceptions, it remained culture positive over the 4 weeks with the number of IFU shed decreasing with time. Therefore while there was significant protection afforded by the INP0341 formulation, once a mouse was infected, regardless of treatment group, infected mice were similar in terms of IFU shed after the first week following infection (P>0.05).

### Effect of the formulation on vaginal tissue

**Figure 7:**
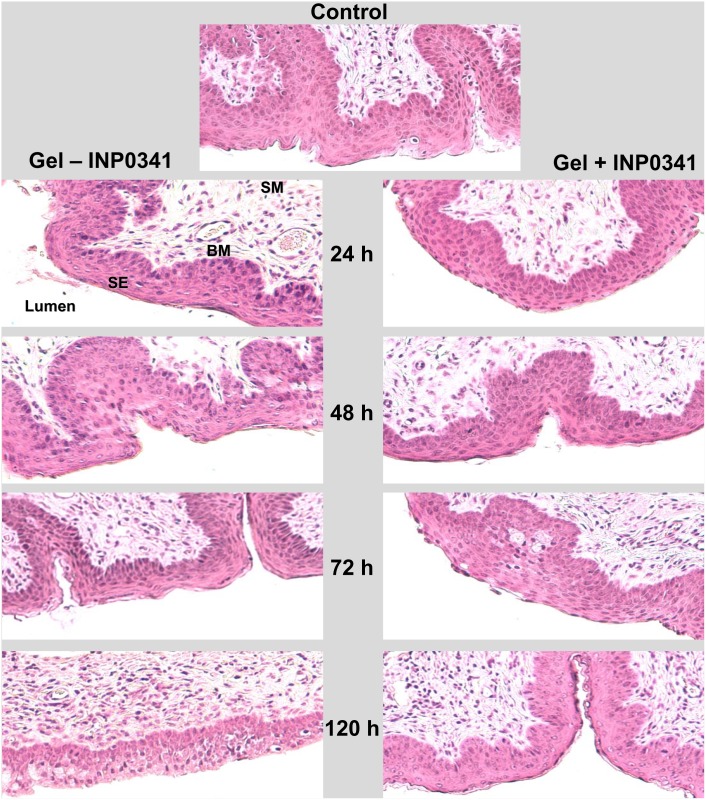
Histopathology of vaginal tissue from mice after gel exposure. Hematoxylin and eosin stained sections of vaginal tissue from mice treated with the PAA vaginal gel formulated without (left panel) and with (right panel) 1 mM INP0341. Mice were treated as described in Material and Methods and sacrificed at various times during the observation period. No differences were noted in the gel treated groups as shown as well as the control group (top) that was not treated with a gel. There was no difference noted between the groups in appearance or thickness of the surface squamous epithelial cell layer (SE), the basement membrane (BM) or the submucosa (SM). There was no sloughing of the superficial epithelial layers and all layers appeared intact. No significant signs of inflammation or blood vessel damage or dilation were seen in either group of mice.

To determine the effect of the formulation with and without INP0341 on vaginal tissue, groups of mice were inoculated vaginally with the formulation with and without incorporation of INP0341, 0.05 ml/dose, at 12 h intervals for 5 days. Non-treated mice were also included as controls. Mice from each group were sacrificed every 24 h up to 5 days. There was little difference in the appearance of the vaginal mucosa throughout the entire vaginal canal in the control mice and with mice treated with the gel with and without INP0341 (Figure 7). In all cases: the stratified squamous epithelium was intact with the same thickness in matching vaginal sections; the basal layer and submucosa were similar; no hemorrhagic lesions or blood vessel dilation was present; and the number of leukocytes, when present, was the same in both groups, with no obvious signs of inflammation in the formulation treated groups. Therefore, the formulated gel did not appear to have any deleterious effects compared to controls on the integrity vaginal tissue as seen by hematoxylin-eosin examination of the tissue.

## Discussion

INP0341 has previously been shown by *in vitro* and *in vivo* experiments to be a promising antibacterial and antiviral agent with activity against common sexually transmitted pathogens [1], [2], [3], [5], [6]. In these reports this compound was used in a liquid form. Therefore the next logical step would be to formulate this compound into a vaginal microbicidal gel that would have the potential to reduce the acquisition and therefore spread of STDs, in particular *C. trachomatis*, the focus of the present investigation.

A challenge to achieve this goal is finding a suitable solvent for the poorly soluble INP0341. The non-ionic surfactant Cremophor ELP (polyoxyl-35 castor oil) was chosen as a solubilizing agent to allow the production of a gel with the target concentration, 1 mM INP0341. Non-ionic surfactants are in general mild to skin and the mucosa, as compared to ionic surfactants, and therefore preferable for use *in vivo* [21]. The use of surfactants in antimicrobial vaginal products however has been questioned, after the disappointing results from the clinical trials of nonoxynol-9 gels. Nonoxynol-9 is also a non-ionic surfactant commonly used in contraceptives, which also has shown anti-HIV effect *in vitro*. However, when nonoxynol-9 gels were tested in clinical trials for protection against vaginal transmission of HIV, it was observed that this surfactant increased the risk of HIV transmission [22]. The increased risk of transmission is most likely due to an irritating effect on the mucosa, thereby facilitating HIV penetration through the epithelium. However, the mucosal irritation caused by surfactants will vary with the type of non-ionic surfactant, and with the length of the hydrophobic tail [23]. In a recent assessment of EpiVaginal tissue, an *in vitro* human vaginal epithelial model for prediction of vaginal irritation, Cremophor ELP was shown to be promising as solubility enhancer for vaginal products [24]. This study showed that 10% Cremophor ELP had a negligible effect on the epithelium, whereas 1% nonoxynol-9 caused irritation of the epithelium [24]. These results suggest that Cremophor ELP is suitable as solubility enhancer in vaginal products. Cremophor ELP was therefore used in the present study, and it was observed that 1.6 wt% Cremophor ELP was sufficient to solubilize 1 mM of INP0341.

We designed a gel based on a mixture of the two polymers Carbopol and Polycarbophil as gelling agents. Both are PAA polymers commonly used in vaginal products [9]. PAA polymers have previously been reported to have intrinsic antimicrobial properties; the Carbopol-based vaginal gel BufferGel was reported to show protection against herpes simplex virus and *C. trachomatis* infections [10]. Another advantage with PAA polymers in vaginal gels, in addition to their inherent antimicrobial properties, is their strong mucoadhesiveness which favors long gel retention times on the mucosal surface [24]. Our aim was to produce a formulation with stronger antimicrobial properties than the PAA gelling agents alone by incorporating an active compound, INP0341, which has been shown in a mouse model to protect from a vaginal infection with *C. trachomatis* [6].

In this work we targeted a gel with yield stress, i.e. a gel for which a stress needs to be applied to enable it to flow, to increase the chance of long contact time with the mucosa and reduce the risk for the formulation to leak out of the vagina. The yield stress of the formulated INP0341, the stress that needs to be applied in order for the gel to flow, was determined to be 11.0 Pa, which was slightly lower than the commercial vaginal gels Crinone (16.2 Pa) and Replens (14.3 Pa). In comparison, the yield stress of a HEC gel, commonly used as a universal placebo gel, is negligible. Yield stress is beneficial for vaginal gels, in the sense that it decreases the risk of gel leakage from the vagina. On the other hand, too high a yield stress value increases the risk that the gel will not spread and cover the whole mucosa. PAA, 1.5 wt%, was chosen as suitable polymer concentration for the INP0341 formulation, since this formulation was similar to the commercial gels Crinone and Replens, regarding yield stress and estimated gel spreading rate (File S1). It was estimated that 83% of the human vaginal mucosa would be covered 2 h after administration of a 1.5 wt% PAA gel. Hence, suggesting that the formulation in future human studies could be applied a few hours prior to sexual intercourse to maximize the protection against STDs.

The osmolality of the 1 mM INP0341 gel was observed to be 658±6.0 mmol/kg. There is a large variation in osmolality among commercial vaginal products, although most products are within the range 100–3,000 mmol/kg [8]. In general, high osmolalities should be avoided since such products possibly can lead to epithelial disruption, although there is no general agreement regarding acceptable osmolality range for vaginal products [8]. The osmolality value of the currently investigated gel is not expected to have any adverse effect on the human vaginal epithelium.

The 1 mM INP0341 gel formulation was relatively stable during storage that is a critical parameter especially when considering distribution and storage limitations in various parts of the world. The fraction of INP0341 degraded after 8 months of storage was observed to be 2.2±5.9% and 5.7±4.2% at 8°C and 20°C, respectively. At an elevated temperature, 40°C, 14.7±2.5% was degraded after 8 months. A decrease in viscosity was also observed after 8 months of storage at 40°C, as shown in Figure 2. The viscosity of the 1.50 wt% PAA gel after 8 months of storage at 40°C was roughly the same as the viscosity of a freshly made 1.25 wt% PAA gel (File S1). It is expected that there should be some decrease in viscosity upon storage, since PAA polymers are subject to oxidative degradation in aqueous solution [19]. The observed decrease in viscosity is however relatively small [7], [18]. Nevertheless, the decrease in viscosity shows that there is a need to include an antioxidant in the final gel product for eventual human use. Ascorbic acid is an example of an antioxidant, commonly used in vaginal products at concentrations 0.01–0.1%, which would limit PAA degradation during storage [9].

Another property that needs to be considered when formulating a microbicide is the effect on the normal vaginal flora. In this study we employed two species of *Lactobacillus* to represent the normal human vaginal flora. Neither the gel with or without INP0341 appeared to inhibit *L. jensenii* or *L. crispatus*. Hence, the results obtained in this study suggest that the normal vaginal flora would not be affected by the formulation.

A well-established mouse model using C3H/HeJ mice was used to test the gel formulation of 1 mM INP0341 since this mouse strain has been shown to be susceptible to a low dose vaginal challenge with a human *C. trachomatis* serovar [5], [11], [12]. Repeated application of the INP0341 containing vaginal gel over 12 h intervals failed to reveal histological changes to the mouse vaginal tissue (Figure 7). Using the same vaginal infection model and prolonged treatment up to 5 days after a vaginal challenge with *C. trachomatis*, there was significant protection in mice treated with the gel (Figure 5). While the gel without INP0341 afforded some degree of protection, the incorporation of INP0341 was proven to increase the protection from this sexually transmitted organism. While the infectious dose of *C. trachomatis* used to challenge the mice was low, 5×10^2^ IFU/mouse, we were able to infect all of the mice inoculated in the positive control, non-treated group. In future studies we plan not only to reduce the number of treatments with the formulated gel but also to challenge mice with higher doses of infectious *C. trachomatis*, in order to further test the level and strength of protection. In addition, in future studies we will investigate the protection of the gel formulation *in vivo* against other STDs such as herpes simplex virus and HIV, which INP0341 has been previously shown *in vitro* to be active against (1,6). INP0341 is indeed particularly interesting for use in microbicidal vaginal gels, since it has both antiviral and antibacterial effects. In the case of protection against HIV in humans no protection is expected from the PAA polymers base formulation (PAA alone) since no such protection was observed in a previous clinical trial of another vaginal gel composed of PAAs [25]. The polymer choice and rheological optimization in this work will, however, maximize mucosal gel coverage and retention time, and the anti-HIV effect of INP0341 should therefore be optimized with the PAA gel formulation presented here.

In summary, we designed a vaginal gel formulation that was able to lend significant protection in a mouse model against intravaginal inoculation of *C. trachomatis* by combining PAA with the microbicidal compound INP0341. This gel formulation has the desired yield stress and rheology for vaginal application and is stable for up to eight months. *In vitro* and *in vivo* studies confirmed that the formulated INP0341 gel lacks toxicity and has the ability to protect from a vaginal challenge with *C. trachomatis*. Future studies will investigate protection by the INP0341 formulation against other STDs such as HIV and herpes simplex virus.

## Supporting Information

File S1 (DOC)
