# Supplementary material for: Formulation of the Microbicide INP0341 for In Vivo Protection against a Vaginal Challenge by Chlamydia trachomatis
Source: PLoS One. 2014 Oct 30;9(10):e110918. doi: 10.1371/journal.pone.0110918 (PMC4214720; doi:10.1371/journal.pone.0110918)
Supplement: File S1 — (DOC) [file pone.0110918.s001.doc]

# “Formulation of the microbicide INP0341 for *in vivo* protection against a vaginal challenge by *Chlamydia trachomatis*”

**SUPPORTING INFORMATION**

The elastic modulus was measured as function of strain for gels with 1.00-2.00 wt% PAA and for the commercial gels Crinone and Replens, as shown in Figure S1. The data obtained was used to calculate yield stress, as described in the Material and Methods section. The yield stress of the INP0341 formulation used *in vivo* was determined to be 11.0 Pa, which was slightly lower than the commercial vaginal gels Crinone (16.2 Pa) and Replens (14.3 Pa), as shown in Table S1. Yield stress is the stress that needs to be applied in order for the gel to flow. Yield stress is beneficial for vaginal gels, in the sense that it decreases the risk of gel leakage from the vagina. Too high yield stress value, on the other hand, increases the risk that the gel will not spread and cover the whole mucosa.

**Figure S1.** Elastic modulus as function of strain, for gels with PAA concentrations of 1.0-2.0 wt% and for two commercial vaginal gels. The measurements were performed by oscillating rotation at a frequency of 1 Hz, at 37°C.

Shear stress was measured as function of shear rate, using rotational viscometry. As shown in Figure S2, the PAA gels exhibited higher shear stresses as compared to Crinone and Replens. Consistency and shear-thinning indices for each gel were obtained by fitting the shear stress data to Eq. 2, as explained in the Materials and Methods section.

**Figure S2.** Shear stress as function of shear rate, for gels with PAA concentrations of 1.0-2.0 wt%. The measurements were performed by continuous rotational viscometry, at 37°C. The lines represent fittings to Eq. 2.

To obtain an estimate for the gel spreading rate over vaginal mucosa, a theoretical squeezing flow model was employed, as previously described . The model presupposes a gel being squeezed between two plates, thereby getting the shape of a disc. The volume of the gel is denoted *V*, the height of the gel is denoted *2h*, the surface area of the gel is approximated as *2πr^2^* (upper and lower circular area of the disc shaped gel) and the gel is being squeezed by a constant force *F*. The model also includes the gel properties yield stress (τ_0_), consistency index (m) and shear-thinning index (n) according to the following:

$\frac{dh}{dt}=-C^{-(1/n)}h^{\frac{3+5n}{2n}}\left[Fh-Qh^{-3/2}\right]^{1/n}$ (Eq. S1)

where

$Q=\left(\frac{\pi\tau_{0}}{3}\right)\left(\frac{V}{2\pi}\right)^{3/2}$ (Eq. S2)

$C=\left(\frac{\pi m}{n+3}\right)\left(\frac{2n+1}{2n}\right)^{n}\left(\frac{V}{2\pi}\right)^{\frac{n+3}{2}}$ (Eq. S3)

The model was used to estimate the part of the vaginal area covered with gel after 120 min, for PAA gels with 1.00-2.00 wt% PAA and for the commercial gels Crinone and Replens. The parameters used (obtained at 37°C), and the resulting area coverage for each gel, are presented in Table S1. Calculations were also made based on literature parameter values (obtained at 23.8°C ± 0.8°C), for the commercial gels Replens, KY Plus and Advantage, as shown in italic script in Table S1.

The part of the vaginal surface area covered with gel after 120 min ranged from 105% (for 1.00 wt% PAA) to 71% (for 2.00 wt% PAA). A value higher than 100 % could be interpreted as leakage of gel from the vagina, but we do not make that interpretation in this work. The model does not take into account factors such as gravity, pH variations, dilution with vaginal fluid, etc., and we merely consider the percentage result a rough estimate that can be used to compare different gels with each other.

Two observations are made regarding the calculations on the commercial gels: (1) Firstly, Replens was found to cover 103% of the vaginal area after 120 min, when using the parameters obtained at 37°C in the present work. When calculations were made for Replens using the literature parameter values, obtained at 23.8°C ± 0.8°C, the result is 75% coverage. It is expected that the gel will spread faster at 37°C, as compared to 23.8°C. (2) Secondly, when comparing the surface coverage results for Replens, KY Plus and Advantage, based on literature parameter values, they vary from 55 to 94%. It can thereby be noted that commercial vaginal gels differ considerably regarding rheological properties, which also has been stated in other work .

The gel formulation with 1.5 wt% PAA was chosen as suitable for the INP0341 formulation, since that formulation was similar to the commercial gels Crinone and Replens, both regarding yield stress and estimated gel spreading rate (see Table S1).

**Table S1.** Prediction of vaginal surface area covered by various gels at different times after administration. A mathematical simulation of squeezing flow was used, as previously described .

| Formulation | Yield stress,  τ_0_  (Pa) | Consistency index, m  (Pa s^n^) | Shear-thinning index, n | Part of vaginal area covered  after 120 min  (%) |
| --- | --- | --- | --- | --- |
| 1.00 wt% PAA | 7.20 | 66.2 | 0.3139 | 105 |
| 1.25 wt% PAA | 7.86 | 82.4 | 0.2863 | 95 |
| 1.50 wt% PAA | 11.0 | 107.3 | 0.2619 | 83 |
| 1.75 wt% PAA | 21.0 | 110.3 | 0.2651 | 76 |
| 2.00 wt% PAA | 26.2 | 115.3 | 0.2520 | 71 |
| Crinone | 16.2 | 36.3 | 0.2921 | 98 |
| Replens | 14.3 | 27.8 | 0.2775 | 103 |
| *Replens* | *41.3* | *9.95* | *0.612* | *75* |
| *KY Plus* | *90.4* | *44.9* | *0.367* | *55* |
| *Advantage* | *22.9* | *15.7* | *0.423* | *94* |

**References**

1. Kieweg, S.L. and D.F. Katz, *Squeezing Flows of Vaginal Gel Formulations Relevant to Microbicide Drug Delivery.* Journal of Biomechanical Engineering, 2006. **128**: p. 540-553.

2. Dezzutti, C.S., et al., *Is Wetter Better? An Evaluation of Over-the-Counter Personal Lubricants for Safety and Anti-HIV-1 Activity.* PLoS ONE, 2012. **7**(11): p. e48328.
